# Molecular basis underpinning MR1 allomorph recognition by an MR1-restricted T cell receptor

**DOI:** 10.3389/fimmu.2025.1547664

**Published:** 2025-03-26

**Authors:** Richard J. Suckling, Cevriye Pamukcu, Robert Alan Simmons, Daniel Fonseca, Emma Grant, Rory Harrison, Saher A. Shaikh, Rahul C. Khanolkar, Hemza Ghadbane, Andrew Creese, Miriam Hock, Thomas G. Gligoris, Marco Lepore, Vijaykumar Karuppiah, Mariolina Salio

**Affiliations:** Immunocore Ltd., Abingdon, United Kingdom

**Keywords:** MR1, cancer, metabolites, MAIT, TCR

## Abstract

**Introduction:**

The MHC-class-I-related molecule MR1 presents small metabolites of microbial and self-origin to T cells bearing semi-invariant or variant T cell receptors. One such T cell receptor, MC.7.G5, was previously shown to confer broad MR1-restricted reactivity to tumor cells but not normal cells, sparking interest in the development of non-MHC-restricted immunotherapy approaches.

**Methods/Results:**

Here we provide cellular, biophysical, and crystallographic evidence that the MC.7.G5 TCR does not have pan-cancer specificity but is restricted to a rare allomorph of MR1, bearing the R9H mutation.

**Discussion:**

Our results underscore the importance of in-depth characterization of MR1-reactive TCRs against targets expressing the full repertoire of MR1 allomorphs.

## Introduction

The major histocompatibility complex (MHC)-class-I-related protein 1 (MR1) presents small microbial metabolites, mostly intermediates of the vitamin B2 biosynthetic pathway (1), to an abundant innate-like T cell population known as mucosal associated invariant T cells (MAIT) (2), bearing a semi-invariant T cell receptor (TCR) (3). Other MR1-restricted T cells with polyclonal TCRs have been described, and they preferentially recognize tumor cells independently of the microbial MAIT ligands (4, 5). Recently, a broadly reactive, MR1-restricted T cell subset with a semi-invariant TCR-α chain incorporating the Jα42 segment was identified in several blood donors (6). While the full range of metabolites recognized by MR1-T cells and the extent of their cancer specificity remains to be elucidated, recognition of nucleobase adducts has recently been demonstrated (7).

The limited polymorphism of MR1 molecules has the potential to be harnessed to develop universal immunotherapies that can be applied broadly across the human population, irrespectively of individual HLA subtypes. At least six MR1 allomorphs have been identified (8), with MR1*01 being the most frequent (71%), followed by MR1*02 (25%), bearing the H17R mutation, outside of the binding groove. In addition to H17R, MR1*04 (allelic frequency of 1%) also bears a mutation in the antigen binding groove, R9H. A homozygous MR1 R9H mutation has been found in an immunodeficient patient, lacking circulating MAIT cells because MR1 R9H is not able to bind and present the selecting antigen 5-OP-RU (9). Other MR1 SNPs are either silent or regulate its expression level and possibly disease susceptibility (10).

In 2020, Crowther and colleagues isolated from the peripheral blood of a healthy donor an MR1-restricted TCR (MC.7.G5) which recognized most human cancer cell lines and primary cancer cells tested, but not normal cell lines or primary cells (5). These results spearheaded the interest in the development of HLA-unrestricted TCR therapies. With the aim to investigate the molecular mechanism of MR1 recognition by the MC.7.G5 TCR, we discovered that rather than reacting to all MR1^+^ cancers, as previously reported, the MC.7.G5 TCR was instead specific for the MR1*04 allomorph and only recognized MR1*01 when over-expressed. In this work, we present cellular, biophysical, and structural evidence underpinning MR1*04 recognition by the MC.7.G5 TCR.

## Materials and methods

### Production of MC.7.G5 TCR

The alpha and beta chains of the MC.7.G5 TCR were expressed in *Rosetta2 E. coli* cells (Novagen) as inclusion bodies, refolded and purified as previously described (11). Codon optimization of the MC.7.G5 TCR alpha chain for *E. coli* improved the expression and subsequent inclusion body purity. A subsequent second size exclusion chromatography run (using S200 10/300 column) (Cytiva, Marlborough, MA, USA) was performed following purification to reduce the presence of free TCR β--chain.

### Production of MR1–ligand complexes

Human MR1 R9H (23-292, C283S) and β-2 microglobulin (21–119) were expressed in *Rosetta2 E. coli* cells (Novagen) using auto-induction media and purified as inclusion bodies as previously described (12). Refolding and complex formation were done by dilution of 60 mg of MR1, 30 mg of β2m, and 2.2 mg 5-FSA (Fluorochem, UK) into 1 L of 0.1 M Tris, pH 8.5, 0.1 M NaCl, 0.8 M L-arginine, 2 mM EDTA, 3.1 mM cystamine, and 7.2 mM cysteamine. After overnight incubation at 4°C, the refold buffer was dialyzed against two changes of buffer containing 10 mM Tris, pH 8.1, and 0.1 M NaCl with at least 24 h between each change. The refolded MR1–β2m–ligand complex was then purified by sequential POROS 50HQ (Thermo Fisher Scientific) anion exchange and size exclusion chromatography (using S75 10/300 column) (Cytiva, Marlborough, MA, USA). 5-(2-oxopropylideneamino)-6-d-ribitylaminouracil (5-OP-RU) was produced by Gurdyal Besra (University of Birmingham, UK), 6-formylpterin (6-FP) and Acetyl-6-formylpterin (Ac-6-FP) were from Schircks Laboratories, and pyridoxal hydrochloride was from Fisher Scientific (for MR1 refolds). For SPR measurements, MR1 with a C-terminal AVI-tag™ was used and biotinylated after purification using BirA (Avidity BirA-500 kit) (according to the manufacturer’s instructions) before being re-purified by size exclusion chromatography and stored at -80°C.

### Production of scMR1-Fc

Soluble MR1 ectodomain was expressed as a single chain construct N-terminally fused to B2M and C-terminally fused to an engineered human Fc domain (hIgG1e3, InvivoGen, San Diego, CA, USA) upon transient transfection of mammalian Expi293F™ cells using Expifectamine, with pCDNA3.1 DNA constructs including C-terminal biotin ligase (AviTag™) and TwinStrep tags (IBA Lifesciences). Expi293F™ cell supernatants were collected after 6 days and clarified by vacuum filtration using a 0.45-µm filter. Following the removal of biotin with BioLock (IBA Lifesciences), protein was purified from culture supernatant using StrepTactinXT™ resin (IBA Lifesciences) and size exclusion chromatography (SEC) (S200 10/300 column). A middle fraction of the SEC peak was enzymatically biotinylated using BirA (Avidity BirA-500 kit) (according to the manufacturer’s instructions) before scMR1-Fc was re-purified by size exclusion chromatography and stored at -80°C.

### Surface plasmon resonance

Purified TCR molecules were subjected to surface plasmon resonance (SPR) analysis using a BIAcore™ T200 (Cytiva, Marlborough, MA, USA). Biotinylated MR1–ligand complexes were immobilized onto a streptavidin-coupled CM5 sensor chip. Flow cell one was loaded with free biotin alone to act as a control surface. All measurements were performed at 25°C in Dulbecco’s PBS buffer + 0.005% P20. Equilibrium dissociation constants (K_D_) were calculated at steady state using GraphPad Prism (GraphPad Software, San Diego, CA, USA) to perform nonlinear curve fit to the data assuming 1:1 Langmuir binding. The dissociation constants (K_D_) were calculated at equilibrium, mean ± SD. All titrations were repeated at least in duplicate in independent experiments.

### Crystallization and structure solution

MC.7.G5 TCR was mixed with MR1 R9H-5-FSA in equimolar ratio, concentrated to 4 mg/mL, and buffer exchanged into 10 mM Tris, pH 8.0, and 20 mM NaCl. Sitting drops were set up containing 150 nL of protein solution and 150 nL of reservoir solution in MRC crystallization plates using the Gryphon robot (ART Robbins) and incubated at 20°C in Rock Imager 1000 (Formulatrix). A single crystal appeared after 3 months in the reservoir solution containing 50 mM HEPES, pH 7.0, 250 mM NaCl, and 11% PEG 8000. The crystal was cryoprotected using a reservoir solution containing 30% ethylene glycol and flash-cooled in liquid N_2_. Diffraction data was collected at beamline I04 at the Diamond Light Source, UK.

The diffraction data were integrated and scaled using the autoPROC processing pipeline (13). The structure was solved by molecular replacement using the MR1 R9H coordinates from PDB 6W9V and TCR coordinates from PDB 7ZT4 as search models in Phaser (14) within the CCP4 suite (15). The model was built using iterative cycles of manual model building in COOT (16) and refinement using Refmac (17). The stereochemical properties and validation of the model was assessed using the PDB-REDO server (18) and Molprobity (19). Data collection and refinement statistics are given in **Table 1**. The buried surface area and TCR docking geometry were calculated using Molecular Operating Environment (Chemical Computing Group). The structural figures were generated using Pymol (Schrödinger).

**Table 1:** X-ray data collection and refinement statistics.

PDB code	9HI7
Molecule	MR1^R9H^-MC.7.G5 TCR
Space group	P 1 21 1
Unit cell dimensions	*a* = 72.03, *b* = 113.20, *c* = 130.36; α = 90°, β = 90.73°, γ = 90°
X-ray source	DLS I04
Wavelength (Å)	0.9795
Resolution range (Å)	130.35–2.81 (2.86–2.81)
Observations	347,251 (17,014)
Unique reflections	51,269 (2,548)
Multiplicity	6.8 (6.7)
Completeness (%)	99.9 (100.0)
Mean I/σ (I)	7.1 (0.6)
*R*_merge_	0.207 (3.03)
*R*_meas_	0.225 (3.287)
*R*_pim_	0.085 (1.264)
CC_1/2_	0.995 (0.382)
Refinement	
*R*_work_/*R*_free_ (%)	23.6/29.6
RMS (bonds)	0.0113
RMS (angles)	1.7911
Mean B value (Å^2^)	60.57
Number of non-hydrogen atoms	13,042
Ramachandran statistics	
Favored (%)	93.16
Allowed (%)	6.78
Outliers (%)	0.06

Values in the parentheses refer to the outer resolution shell.

### Cells

THP1 and C1R cells were transduced with full-length MR1*01 (20), scMR1*01, scMR1 H17R, scMR1 R9H, or scMR1 R9H H17R (9); THP1 β2m KO were generated by using CRISPR Cas9 (21). A549 WT and MR1 KO were a gift from Prof. David Lewinsohn, Portland, OR, USA.

NK92 cells (ATCC) were transduced with viruses encoding for each of the CD3 subunits (delta, gamma, epsilon without zeta) (22), the MC.7.G5 TCR (5), sorted, and used for *in vitro* recognition assays.

The growth media were RPMI 10% FCS for C1R/THP1 and DMEM 10% FCS for A549. The media were supplemented with glutamine, pen/strep, non-essential amino acids, sodium pyruvate, and HEPES (all tissue-culture-grade, Gibco). For NK92, we used Gibco α-MEM, without nucleosides, and supplemented with 12.5% hi FBS, 12.5% horse serum, 2mM L-glutamine/GlutaMAX, 0.1 mM 2-mercaptoethanol, 0.2 mM inositol, 0.02 mM folic acid.

### Functional assays

Stimulation with target cell lines at E:T 2:1 was performed in duplicate in 96-well plates in 200-μL medium. 5 × 10^5^ effectors were used per well, and for NK92 cells CD107a/CD107b APC antibodies (Miltenyi) were used during the incubation. After 1 h of co-incubation, Monensin solution 1000X (Biolegend) was added at a 1:500 dilution. Where indicated, the target cells were pre-incubated 4 h or overnight with 10 μM 5-OP-RU, 10 μM Ac-6-FP, 100 μM 5-FSA, or 100 μM pyridoxal (20). The anti-MR1 26.1 antibody (Biolegend) was used at 20 μg/mL in blocking experiments, with a relevant isotype control (Biolegend). Effector cell activation was assessed by flow cytometry after 4 h (for NK92) or overnight (for Jurkat cells). Cells were harvested, stained with a live/dead dye (either Aqua or near IR, both from Biolegend) and with CD69 PerCPCy5.5 (Biolegend, for Jurkat only), and acquired on a Fortessa X20 equipped with four lasers. Analysis was performed with FlowJo v10 (TreeStar Inc). MR1 staining was performed with the 26.5-PE antibody (Biolegend) or the IgG2a-PE isotype control (Biolegend).

Plate bound assays were performed as described (23).

### Sequencing

Genomic DNA was extracted from the A549 cell line using QuickExtract DNA kit (Cambio Ltd., Cambridge, UK). MR1 locus (exon 2) was amplified from the DNA using PCR with AmpliTaq Gold360 (ThermoFisher Scientific, UK) (following the manufacturer’s instructions) and the following primers: P1F 5′-CTGAGTAGGGAGCACTCGT or P2F 5′-CTGGATCATCTGGGACCCTA and P1-2R 5′-AAATGGGATGCAGATACGGATA. The PCR products were cleaned and sent for Sanger sequencing with Genewiz using the abovementioned primers.

### xCELLigence assay

Real-time cell viability experiments were performed using the xCELLigence eSight device (ACEA Biosciences) placed in a humidified incubator at 37°C with 5% CO_2_. First, E-Plate 96 plates were pre-incubated with 100 μL of cell-free growth medium containing 10% fetal bovine serum (FBS) for 30 min to equilibrate the temperature. After this incubation, a background impedance signal was measured to confirm all connections.

Target cells, including A549 WT and A549 MR1 KO cells, were seeded at a density of 1.5 × 10^4^ cells per well in 100 μL of medium. The plates were then incubated at room temperature for 30 min to allow the cells to settle before being transferred to the xCELLigence device for continuous monitoring. The target cells were cultured for approximately 24 h until the cell index (CI) reached at least 1, before the addition of effector cells. Effector cells were added the next day at an effector-to-target (E:T) ratio of 1:1. Real-time measurements of cell viability were recorded every 15 min for 70 h by monitoring the cell index (CI) using the RTCA software for data analysis. Specific lysis at 10 h was calculated as follows: [(CI_effector+ targets_)/CI_targets_] × 100.

## Results

### MC.7.G5 TCR preferentially recognizes MR1 R9H

**Figure 1:**
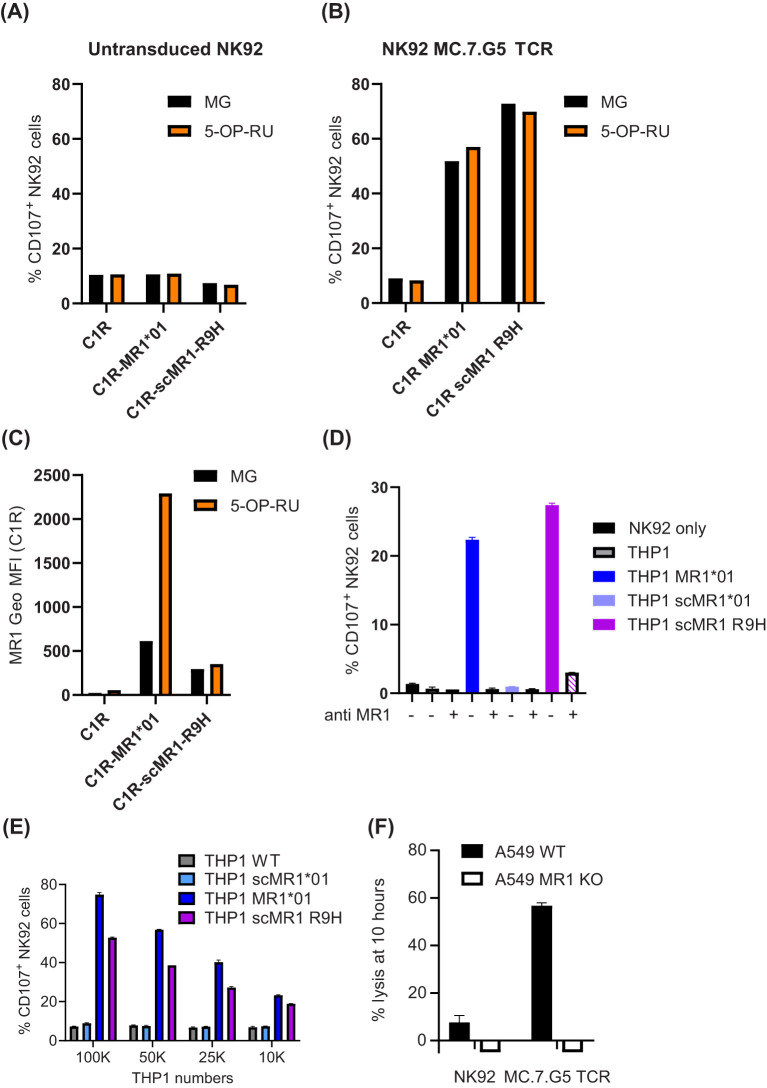
NK92 cells transduced with the MC.7.G5 TCR preferentially recognize MR1 R9H targets. **(A–C)** C1R cells WT, over-expressing MR1*01 or scMR1 R9H and unpulsed (black bars) or pulsed 4 h with 10 μM 5-OP-RU (orange bars), were incubated with NK92 untransduced **(A)** or transduced with the MC.7.G5 TCR **(B)**. NK92 activation was measured after 4 h and is depicted as % of CD107^+^ cells. Geometric mean fluorescence expression of MR1 measured by flow cytometry is depicted in **(C)**. **(D)** NK92 MC.7.G5 cell activation in response to THP1 WT, transduced with MR1*01, scMR1*01, or scMR1 R9H, in the presence or absence of the blocking MR1 antibody 26.5 (20 μg/mL). **(E)** NK92 MC.7.G5 cell activation in response to decreasing numbers of THP1 WT, transduced with MR1*01, scMR1*01, or scMR1 R9H. **(F)** Killing of A549 WT (black bars) or MR1 KO cells (white bars) by NK92 MC.7.G5 cells, measured by xCELLigence assay at 10 h.

To understand the ligand specificity of the published pan-cancer MR1-restricted T cell clone, MC.7.G5 (5), we transduced the TCR in Jurkat cells lacking endogenous TCRβ and in the NK92-CD3 cytotoxic cell line. We did not observe any activation of NK92-MC.7.G5 cells (% of CD107^+^ cells) in response to C1R or THP1 (which have endogenous MR1), unless full-length MR1*01 was over-expressed at non-physiological levels (**Figures 1A–E**, **Supplementary Figure S1A**). However, we did observe cytolytic activity when NK92-MC.7.G5 cells were incubated with A549 WT but not MR1 KO (**Figure 1F**). We therefore sequenced the A549 cell line, against which the MC.7.G5 clone was originally identified (5), and observed that it is heterozygous for the MR1*04 allele (8), containing both R9H and H17R mutations (**Supplementary Figure S1B**). While the H17R mutation sits outside of the binding groove and is unlikely to affect ligand binding (**Supplementary Figure S1C**), the R9H mutation has been shown to impair the presentation of the MAIT cell agonist 5-OP-RU (9). We next transduced C1R and THP-1 with a single-chain construct encoding for MR1 R9H (scMR1 R9H) and observed NK92-MC.7.G5 activation (**Figures 1A–E**), which could be specifically blocked with the anti-MR1 26.5 antibody (**Figure 1D**). It is interesting to note that despite having a lower surface expression than MR1*01, scMR1 R9H elicited comparable activation of NK92-MC.7.G5 cells (**Figures 1B–D**, **Supplementary Figure S1A**). Additionally, we observed a dose-dependent response when titrating the THP1 numbers in the assay (**Figure 1D**). Next, we investigated whether NK92-MC.7.G5 activation in response to MR1*01 or scMR1 R9H was altered when targets were pulsed with the MAIT Schiff-base forming ligand, 5-OP-RU (**Figure 1B**). In agreement with published data (9), we observed that only MR1*01 but not MR1 R9H was able to present 5-OP-RU, as inferred by cell surface increased expression (**Figure 1C**). Despite a fourfold increase in MR1*01 expression (**Figure 1C**), the reactivity of NK92-MC.7G5 was not increased (**Figure 1B**), consistent with lack of 5-OP-RU reactivity. The reactivity of NK92-MC.7.G5 cells to 5-OP-RU-pulsed scMR1 R9H molecules was also unaffected, consistent with lack of 5-OP-RU binding to MR1 R9H (**Figures 1B, C**).

**Figure 2:**
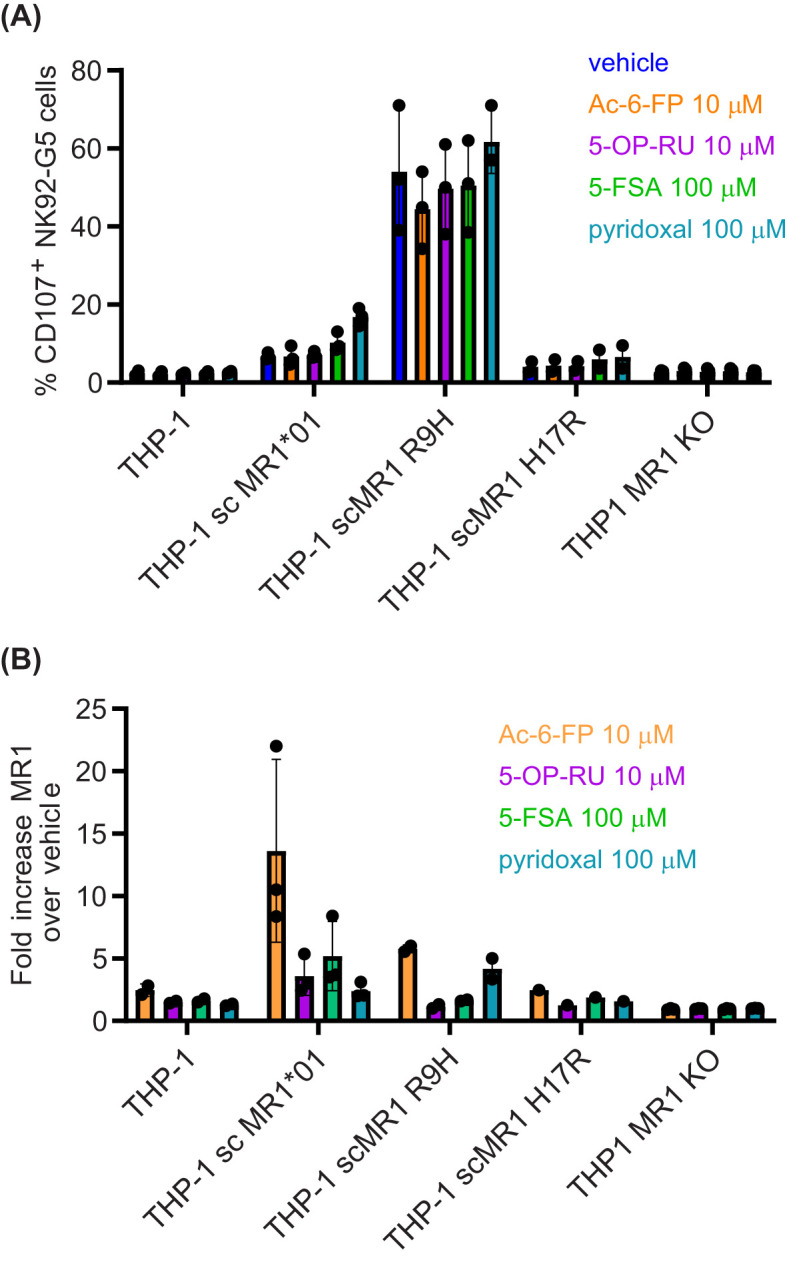
MR1 R9H presents pyridoxal to NK92 MC.7.G5 cells. **(A)** THP-1 cell WT, over-expressing scMR1*01, scMR1 R9H, scMR1 H17R, or THP1 MR1 KO, were pulsed overnight with vehicle, Ac-6-FP (10 μM), 5-OP-RU (10 μM), 5-FSA (100 μM), or pyridoxal (100 μM). On the next day, the cells were washed and **(A)** incubated with NK92 MC.7.G5 cells for a functional assay or **(B)** stained for MR1 surface expression. **(A)** Percentage of CD107^+^ NK92 MC.7.G5 cells. Each point represents the average of duplicates of one biological replica. **(B)** Fold increase MR1 expression (GeoMFI) over basal MR1 expression. Each point represents one biological replica. Representative histogram plots are shown in **Supplementary Figure S2**.

Lastly, we expressed MR1*01 as single-chain molecules for a better comparison to scMR1 R9H (**Figures 1D, E**), and we observed that despite a similar expression at the cell surface (**Supplementary Figure S1A**), THP1 scMR1*01 elicited minimal activation of NK92-MC.7.G5 cells above the background and only at high cell numbers. However, we observed the activation of NK92-MC.7.G5 cells when THP-1 scMR1*01 targets were pulsed with a high concentration of the ligands 5-FSA and pyridoxal (24), but not Ac-6-FP (**Figure 2A**). Non-significant NK92-MC.7.G5 cell activation over the background was elicited by THP-1 scMR1 H17R (likely because it was expressed only on a fraction of the targets; **Figure 2A**, **Supplementary Figure S2A**). A non-significant increase of NK92-MC.7.G5 cell activation was induced by pyridoxal-pulsed THP-1 scMR1 R9H targets, while Ac-6-FP pulsing consistently reduced the activation, although the finding was not statistically significant (**Figure 2A**). All ligands tested induced MR1 upregulation at the cell surface of THP-1 and C1R cells (**Figure 2B**, **Supplementary Figures S2A–C**), although to a different extent. Accordingly, we observed that pyridoxal (vitamin B6) induced preferential upregulation of scMR1 R9H compared to scMR1*01 (24). These results altogether suggest that the TCR MC.7.G5 has preferential reactivity to MR1 R9H. The observed quantitative differences in the expression level of MR1 R9H between C1R and THP1 also suggest that there may be specific ligands, such as pyridoxal, facilitating MR1 R9H transport to the cell surface.

To corroborate these data, MR1 molecules were expressed in Expi293F™ cells as a single-chain soluble molecule N-terminally fused to β2M and C-terminally fused to an Fc domain (scMR1-Fc-endo). In this system, MR1 is secreted as a soluble molecule having been loaded with endogenous ligands (“endo” scMR1-Fc). MR1 molecules containing mutations R9H or K43A (which has been shown to prevent Schiff base formation with ligands (25)) were expressed alongside wild-type MR1*01. Recombinant purified MR1 molecules were immobilized on plates prior to overnight culture with Jurkat cells expressing either a canonical MAIT TCR (VT001) (26), the self-reactive MAIT E8 TCR (26), or the MC.7.G5 TCR. As expected, the Jurkat cell line expressing the canonical MAIT TCR was only activated (% CD69^+^ cells) by 5-OP-RU-pulsed scMR1-Fc-WT*01 molecules, the self-reactive Jurkat-E8-TCR cell line was activated in all conditions, whereas the Jurkat-MC.7.G5 cell line was only activated by scMR1-Fc-R9H (**Supplementary Figures S1D, E**). These results further validate the observation that the MC.7.G5 TCR is preferentially activated by MR1-R9H.

### Biophysical properties of MC.7.G5 TCR

**Figure 3:**
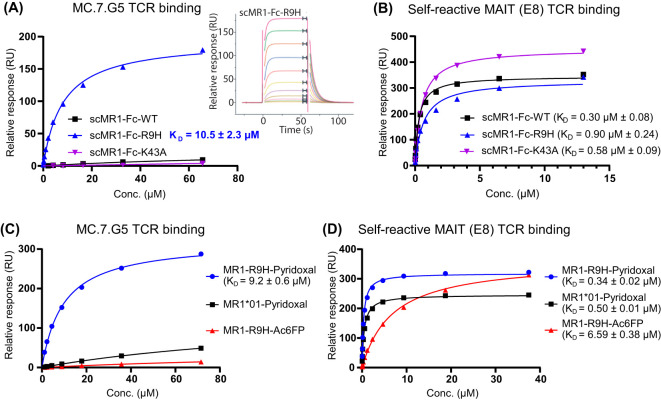
MC.7.G5 TCR binds to scMR1-Fc-R9H and MR1-R9H-pyridoxal. **(A)** Saturation plot and sensorgram (inset) showing the selective binding of MC.7.G5 TCR to scMR1-Fc-R9H (*K*_D_ = 10.5 ± 2.3 µM) as measured by surface plasmon resonance (SPR) (at 25°C). **(B)** Binding of a self-reactive MAIT (E8) TCR to the same sensor chip containing the immobilized scMR1-Fc constructs, indicating that all scMR1 constructs are functional. **(C)** Equilibrium binding of the MC.7.G5 TCR to refolded MR1-R9H-pyridoxal (*K*_D_ = 9.2 ± 0.6 µM) as measured by SPR. Minimal binding (*K*_D_ > 100 µM) is seen towards the MR1*01 pyridoxal complex, and the MC.7.G5 TCR does not bind to MR1-R9H-Ac-6-FP. **(D)** Binding of a self-reactive MAIT TCR (E8) to the same sensor chip containing the refolded MR1–ligand complexes, indicating that all complexes are functional and loaded to a similar level. Dissociation constants (*K*_D_) calculated at equilibrium, mean ± SD. All titrations were repeated in triplicate in independent experiments; one representative titration is shown.

To further investigate the MC.7.G5 TCR specificity, refolded soluble MC.7.G5 TCR molecules were tested for binding to recombinant scMR1-Fc protein constructs expressed in Expi293F™ mammalian cells using surface plasmon resonance (SPR). Injection of the MC.7.G5 TCR over immobilized scMR1-Fc (WT*01, R9H and K43A) showed binding only to scMR1-Fc-R9H with a *K*_D_ of 10.5 ± 2.3 μM (*n* = 3) (**Figure 3A**). For these experiments, approximately 1,000 response units (RU) of scMR1-endo were coupled to the streptavidin SPR chip via the C-terminal biotinylated Avi-tag™ on scMR1, and the maximal response achieved was ~200 RU. Considering ILT2 c50 binding and the molecular weight (27), the abovementioned result indicates that the MC.7.G5 TCR binds to ~40% of the MR1 molecules loaded with an endogenous repertoire of metabolites. Minimal binding was seen toward scMR1-Fc-WT*01, confirming selectivity toward R9H molecules. The MC.7.G5 TCR did not bind scMR1-Fc-K43A molecules, consistent with previous studies (5, 28). Binding of the self-reactive MAIT TCR, E8, to all of the scMR1-Fc constructs showed that they were properly folded (**Figure 3B**). The A549 MR1*04 allelic variant also contains the H17R mutation, a residue distal from the antigen binding pocket. Therefore, binding of the MC.7.G5 TCR to scMR1 containing both the R9H and H17R mutations was also assessed. The MC.7.G5 TCR bound to the scMR1-Fc-R9H-H17R double mutant with a similar affinity to scMR1-R9H as measured by SPR (*K*_D_ = 13.7 vs. 10.5 μM), indicating that the R9H mutation is the key determinant for binding (**Supplementary Figure S3A**). Consistently, minimal binding of the MC.7.G5 TCR (*K*_D_ > 100 μM) was observed towards scMR1-Fc-H17R (MR1*02), as already observed towards scMR1-Fc-WT*01 (**Supplementary Figure S3A**). In addition, the MC.7.G5 TCR did not bind to any refolded MR1*01–ligand complexes by SPR, including MR1-5-OP-RU, MR1-6-FP, or MR1-5-FSA (**Supplementary Figure S3B**). In agreement with recent results (24), we observed that the binding of the MC.7.G5 TCR to MR1-R9H molecules refolded with pyridoxal (vitamin B6), with a *K*_D_ of 9.2 ± 0.6 μM (*n* = 3), with minimal binding (*K*_D_ >100 μM) toward MR1*01–pyridoxal molecules (**Figure 3C**). Binding of the self-reactive MAIT TCR, E8, to the refolded molecules showed that they were all properly folded (**Figure 3D**). No binding was seen to refolded MR1-R9H-Ac-6-FP, consistent with its inhibitory effect on NK92-MC.7.G5 cell activation (**Figures 2A**, **3C**) (24, 29).

### Crystal structure of the MC.7.G5 TCR–MR1 R9H complex

**Figure 4:**
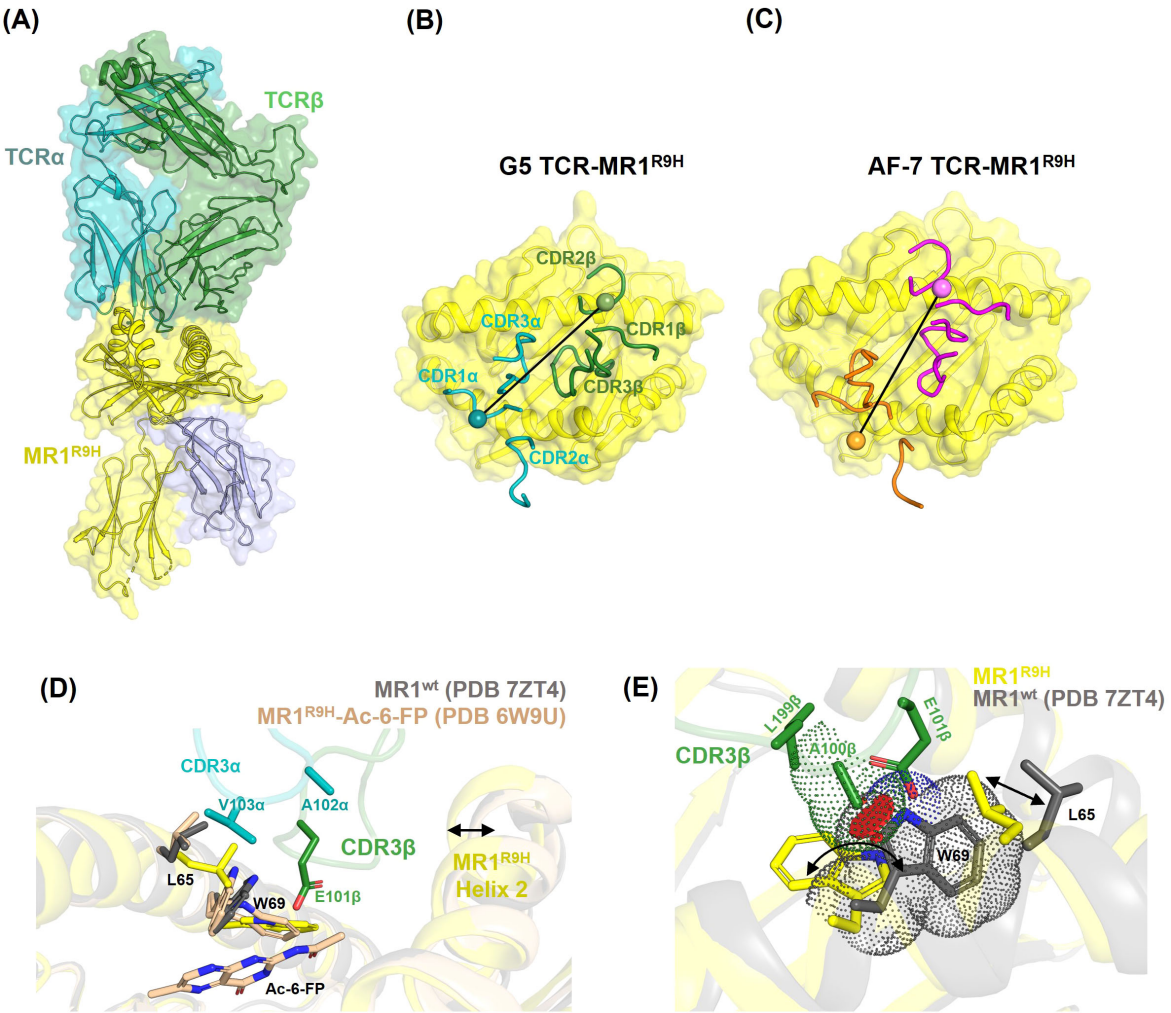
Structure of the MC.7.G5 TCR–MR1 R9H complex. **(A)** Overall view of the complex. MR1 R9H (yellow), β2m (blue), TCRα (cyan), and TCRβ (green) are shown as cartoons. **(B, C)** Top view showing the position of CDRs (cartoon tubes) on the MR1 R9H surface for the MC.7.G5 and AF-7 TCRs, respectively. The position of disulphide bonds on the TCR alpha and beta variable domains are shown as blobs with a line connecting them representing the vector used for crossing angle determination. **(D)** Overlay of the MC.7.G5-TCR-MR1 R9H, MR1*01 (PDB 7ZT4, in gray), and MR1 R9H-Ac-6-FP (PDB 6W9U, in wheat) structures. The long CDR3β of the MC.7.G5 TCR is positioned well into the MR1 R9H groove and pulls the helix2 toward it (indicated by an arrow). CDR3β E101β forms H-bond with MR1 R9H W69 and is within interacting distance to Ac-6-FP in MR1 R9H-Ac-6-FP. **(E)** Overlay of MC.7.G5-TCR-MR1 R9H and MR1*01 (PDB 7ZT4, in gray) structures. The arrows indicate the conformational differences in MR1 residues W69 and L65. The red discs indicate potential clashes between the MC.7.G5 TCR A100β and the MR1*01 W69 residues.

To decipher the molecular basis of MC.7.G5 TCR interactions with MR1 R9H, we solved the structure of the MC.7.G5 TCR in complex with refolded MR1 R9H-5-FSA to a resolution of 2.81 Å (**Table 1**). The MC.7.G5 TCR bound to the MR1 R9H with a docking angle of 43° and buried a surface area of 1,150 Å^2^, with the TCRβ chain contributing the most (60%) (**Figures 4A, B**). All six CDRs were involved in binding, with CDR3β dominating the interactions (**Supplementary Figure S4**). These features differ from the published AF-7-MR1 R9H complex structure (**Figure 4C**). Despite being refolded in the presence of the drug derivative 5-FSA (30), the electron density for the ligand was lacking in the MC.7.G5 TCR–MR1 R9H complex structure, indicating that the ligand was either at a very low occupancy or that MR1 R9H was refolded without a ligand. It is worth noting that MR1 R9H was previously crystallized without the presence of a ligand (9), clearly differing from MR1*01 in the requirements for stable purification. Our crystal structure showed that the long CDR3β loop in the MC.7.G5 TCR reaches deep into the MR1 R9H ligand pocket (**Figure 4D**). When compared to the MR1*01 structures, W69 in MR1 R9H adopted a flipped conformation (**Figure 4E**), as observed previously in the AF-7-MR1 R9H complex structure (9). CDR3β L99β and A100β interacted with MR1 R9H W69 through van der Waals contacts, and the E101β side chain formed a hydrogen bond with the amine group of the W69 side chain (**Figures 4D, E**, **Supplementary Table S1**). These interactions with the flipped conformation of W69 formed the basis of higher specificity of the MC.7.G5 TCR toward MR1 R9H.

When the MR1 R9H–MC.7.G5 TCR complex was overlayed with the MR1*01 crystal structure, the distance between the CDR3β A100 Cβ atom and the W69 side chain was reduced from 3.82 Å in MR1 R9H to 1.7 Å in the MR1 *01 conformation (**Figure 4E**). This shortened interacting distance would potentially introduce steric clashes between CDR3β and W69 in MR1*01 and may explain the lower affinity of MC.7.G5 TCR for MR1*01.

## Discussion

Cancer immunotherapy has come of age, and technological developments in the antibody field spearheaded the development of off-the-shelf therapeutics (31). In view of the quasi-monomorphic nature of the MHC-class I antigen-presenting molecule MR1, it represents an attractive non-MHC-restricted target for T cell redirection. We therefore investigated the molecular mechanisms underpinning its binding to the MC.7.G5 TCR, previously reported to be pan-cancer-reactive while sparing normal cells (5). Unexpectedly, we observed that the MC.7.G5 TCR was not pan-MR1 reactive but was instead specific for the MR1*04 allomorph. Indeed we did not observe the activation of MC.7.G5-transduced cells in response to common myeloid or lymphoid tumor cells which expressed the most frequent MR1 allele, MR1*01. Reactivity to MR1*01 was observed only when the molecules were expressed at non-physiological levels. These results are consistent with a recent report (29) that characterized in detail the reactivity of MC.7.G5-transduced cells to a panel of 133 healthy and cancerous cell lines: reactivity was observed only to MR1*04 targets, irrespective of their normal or malignant origin. The allomorph MR1*04 bears two mutations, R9H, which has been shown to affect the binding of the MAIT cell ligand 5-OP-RU (9), and H17R, which is distal from the antigen-binding groove and does not directly influence the antigen presentation: accordingly, we demonstrated comparable binding of the MC.7.G5 TCR to both MR1*04 and MR1 R9H molecules.

In addition to cell activation assays, we provided biophysical evidence of MC.7.G5 TCR binding to MR1 R9H but not to MR1*01 presenting either the canonical MAIT TCR antigens 5-OP-RU or the known MR1 binders 6-FP or 5-FSA. Furthermore, our high-resolution crystal structure of the TCR-MR1 complex provides the molecular mechanism for the preferential binding of the MC.7.G5 TCR to MR1 R9H: the long TCR CDR3β loop binds deeply in the antigen binding groove of MR1 R9H, with a network of stabilizing interactions with residue W69, which is flipped compared to its position in MR1*01, where it prevents tight binding of the MC.7.G5 TCR. The flipped conformation of W69 in MR1 R9H is likely facilitating the refolding of the molecules in the absence of any ligand, although with lower thermal stability (9). Despite refolding MR1 R9H with 5-FSA, indeed we did not detect any electron density in the antigen binding groove. We cannot however exclude the low occupancy of 5-FSA, and it is possible that at the cell surface MR1 R9H might present a different set of metabolites from MR1*01, eliciting a stronger activation of T cells displaying the MC.7.G5 TCR. In addition, the MC.7.G5 TCR only bound to ~40% of MR1 R9H molecules loaded with endogenously presented metabolites, indicating some ligand specificity. Consistently, the residue R9 has been previously shown to be important for the presentation of the weak agonist diclofenac and its derivative OH-diclofenac (30). It is interesting to note that a flipped conformation of W69 and remodeling of the MAIT TCR CDR3β were also observed in the crystal structure of the AF-7 MAIT TCR bound to MR1*01 in complex with diclofenac and OH-diclofenac (30). Therefore, ligand-dependent W69 flipping can occur in the context of MR1*01. While we have excluded the recognition of 5-OP-RU-bound MR1*01 complexes, activation of the original MC7.G5 TCR was shown to be abrogated by K43 mutations, suggesting dependence on an alternative Schiff-based ligand (5, 25). The observed binding of the MC.7.G5 TCR to MR1-R9H molecules refolded with pyridoxal (vitamin B6), with minimal binding toward MR1*01–pyridoxal molecules, underscores the key role of the R9H mutation in enabling the binding of the MC.7.G5 TCR and suggests that pyridoxal is likely the predominant metabolite loaded in scMR1-Fc-R9H mammalian molecules. The preferential binding of pyridoxal to MR1 R9H molecules over MR1*01 is reflected by an increased half-maximum melting temperature (*T*_m_) of the refolded MR1-R9H–pyridoxal (*T*_m_ = 57.8 ± 0.09°C for MR1 R9H vs. 51.6 ± 0.09°C for MR1*01 (data not shown)) (24). Metabolomic analysis will be required to elucidate the breadth of ligands bound to MR1 allomorphs.

In conclusion, our results highlight the functional relevance of the limited MR1 polymorphism, which needs to be considered when developing MR1-based immunotherapeutic strategies.

## Author’s note

While this manuscript was under internal review, a paper describing the activation of a T cell line transduced with the MC.7.G5 TCR by pyridoxal (vitamin B6) presented by both MR1*01 and MR1-R9H was published (24). As stated in the *Materials and Methods* section, we refolded MR1-R9H with 5FSA, which was not mapped in the crystal structure.

## Data Availability

The datasets presented in this study can be found in online repositories. The names of the repository/repositories and accession number(s) can be found in the article/**Supplementary Material**.
