# Supplementary material for: Molecular basis underpinning MR1 allomorph recognition by an MR1-restricted T cell receptor
Source: Front Immunol. 2025 Mar 26;16:1547664. doi: 10.3389/fimmu.2025.1547664 (PMC11979126; doi:10.3389/fimmu.2025.1547664)
Supplement: Supplementary file 1 [file DataSheet1.docx]

**Supplementary material**

**Supplementary table 1:** List of interactions between MR1 R9H and MC.7.G5 TCR within distance. H-bonds (HB) / salt bridges (SB) are indicated.

| **MR1-R9H** | **G5 TCR** | |
| --- | --- | --- |
| Arg41 | Glu56β | 1SB |
| Arg61 | Glu34α | 2, 3SB |
|  | Ser101α  Val103α  Asn104α | 2, 1HB  1  6 |
| Gln64 | Tyr50β | 7, 2HB |
|  | Glu56β | 1, 1HB |
| Leu65 | Val103α  Tyr50β  Ala100β | 1  1  1 |
|  | Glu101β | 2 |
| Arg67 | Glu56β | 1 |
| Gly68 | Tyr50β | 7 |
|  | Ala100β | 1 |
| Trp69 | Leu99β | 3 |
|  | Ala100β  Glu101β | 8  4, 2HB |
| Gln71 | Tyr50β  Val52β | 1  2, 1HB |
|  | Ser54β | 3, 1HB |
| Met72 | Lys31β | 1 |
|  | Arg97β | 1 |
|  | Leu99β | 3 |
|  | Ala100β | 1 |
| Arg94 | Leu99β | 1 |
| Trp143 | Gly98β | 2 |
| Asn146 | Glu95β  Ala96β | 1, 2HB  1 |
| His148 | Arg55α  Tyr59α  Glu95β | 8, 3HB  1HB  1, 1SB |
|  | Thr103β | 3, 1HB |
| Glu149 | Ala96β | 3 |
|  | Arg97β  Gly98β  Leu99β  Thr103β | 1, 1SB  7, 2HB  5, 1HB  1, 1HB |
| Leu151 | Tyr59α | 3 |
| Tyr152 | Asp36α | 3 |
|  | Ala102α  Glu101β  Thr103β | 1  4  2 |
| Gln153 | Leu99β | 5 |
| Asn155 | Asp36α | 1, 2HB |
| Glu160 | Glu34α | 1HB |
|  | Ser35α | 2, 2HB |
|  | Asp36α | 7, 1HB |

**Supplementary Figure 1**

**
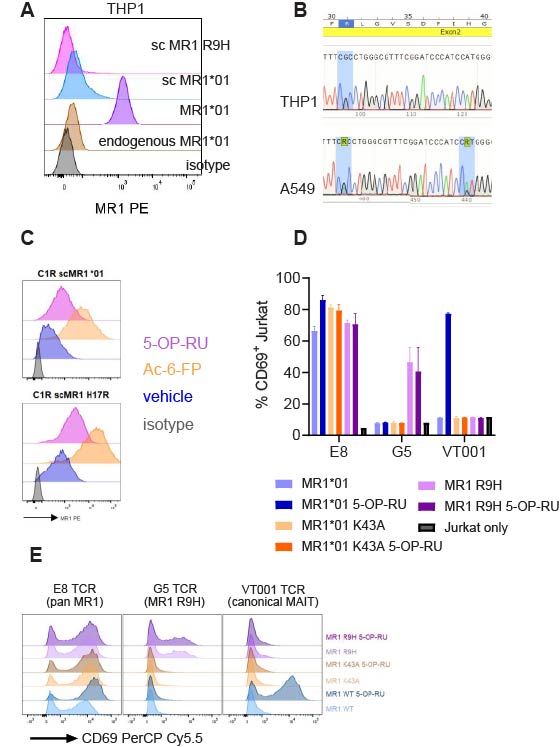
**

**Figure S1.** A. Surface MR1 expression in the THP1 cell lines used for the assays described in Figure 1. B. Sanger sequencing traces of the *MR1* gene in THP1 (allele *MR1*01*) and A549 (heterozygous *MR1*01/MR1*04*) cells. Shaded blue boxes indicate mutations in the *MR1*04* allele. C. Surface MR1 expression in C1R cells expressing scMR1*01 or scMR1 H17R, after overnight pulsing with 5-OP-RU (purple), Ac-6-FP (orange) or vehicle (blue). Gray histograms represent isotype control staining. D and E. Plate bound assay. Activation of Jurkat cells transduced with the E8, MC.7.G5 or VT001 TCRs in response to the indicated MR1 alleles and in the presence or absence of 10 μM 5-OP-RU. D. Mean and standard deviation of duplicates. E. Representative FACS histogram plots.

**Supplementary Figure 2**
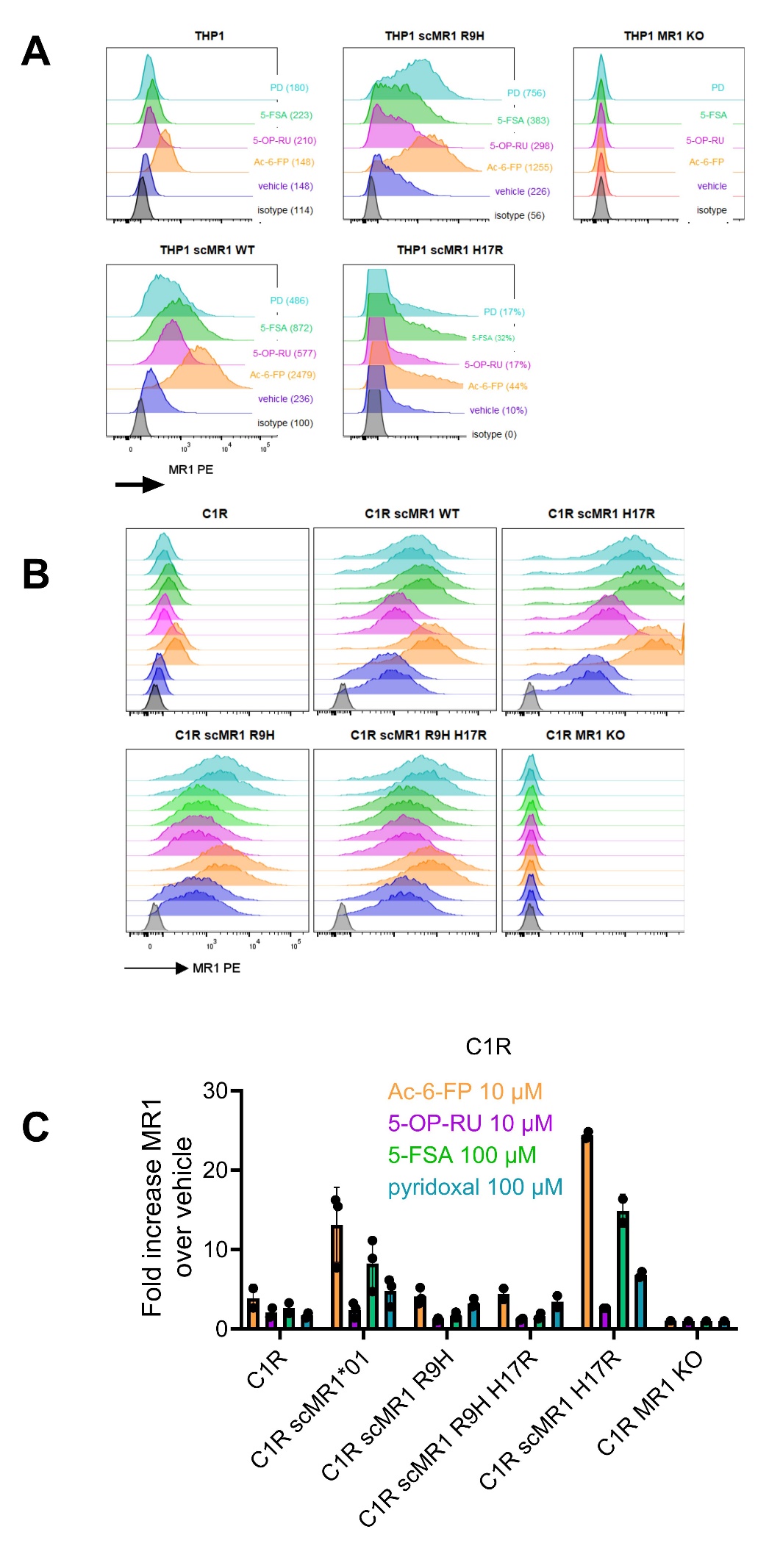


**Figure S2. Ligand binding to MR1 allomorphs.** Cells (THP1, A) or C1R (B, and C) expressing endogenous MR1*01, scMR1*01, scMR1 R9H, scMR1 H17R, scMR1 R9H H17R (C1R only) or MR1 KO, were pulsed overnight with vehicle, Ac-6-FP (10μM), 5-OP-RU (10μM), 5-FSA (100μM) or pyridoxal (100μM). The next day cells were washed and stained for MR1 surface expression. A) THP1 cells, representative histograms profiles for MR1 expression. GeoMFI are indicated in brackets for each treatment. For THP1 scMR1 H17R percentage of MR1 positive cells is plotted. One experiment representative of three; cumulative data are shown in Figure 2. B) C1R cells, representative histograms profiles for MR1 expression. C) Fold increase MR1 expression (GeoMFI) over basal MR1 expression for C1R cells. Each point represents one biological replica.

**Supplementary Figure 3**


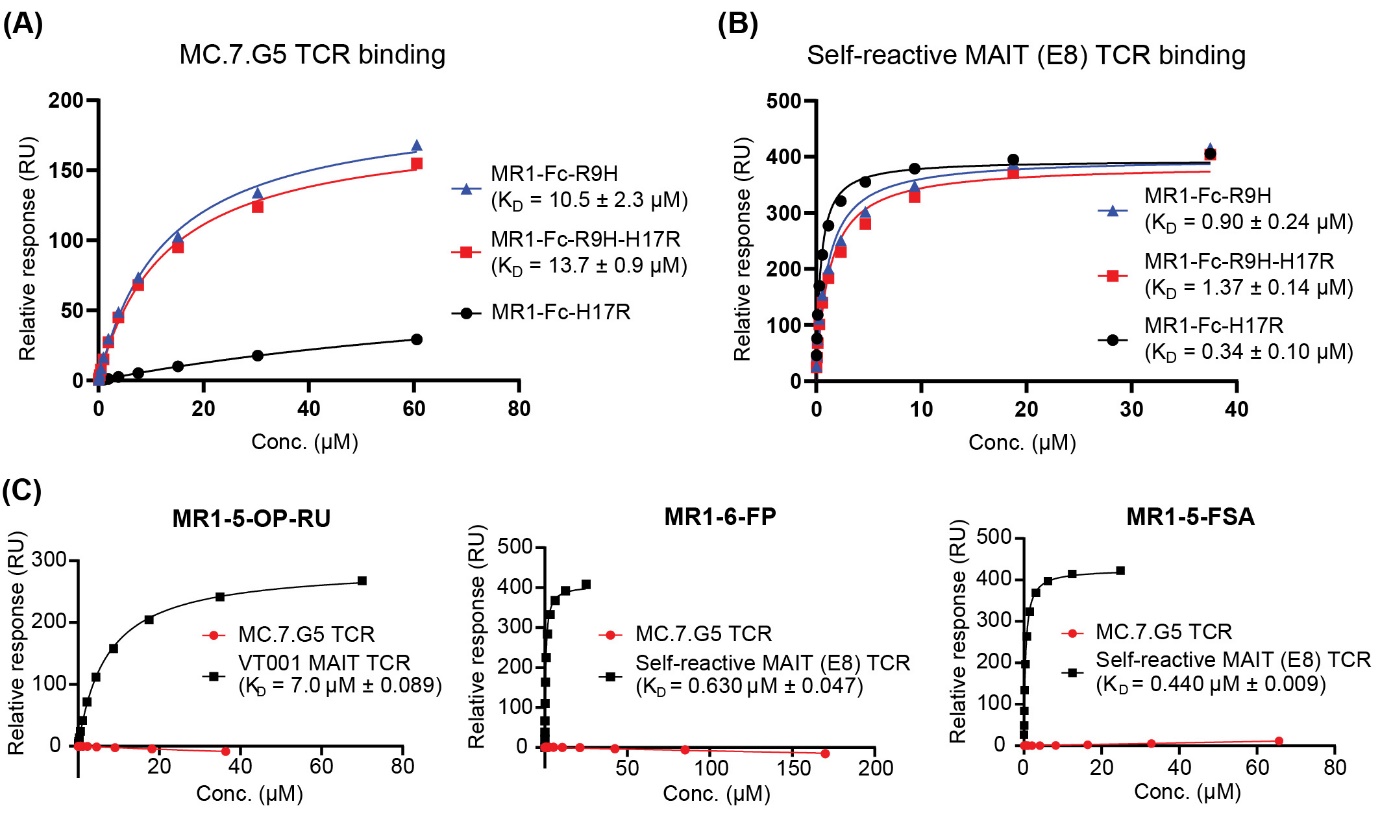


**Figure S3. MC.7.G5 TCR binds to the MR1*04 allele.** A. Equilibrium binding of the MC.7.G5 TCR to both scMR1-Fc-R9H (K_D_ = 10.5 ± 2.3 µM) and scMR1-Fc-R9H-H17R (corresponding to the MR1*04 allele) (K_D_ = 13.7 ± 0.9 µM) as measured by surface plasmon resonance. Minimal binding (K_D_ > 100 µM) is seen towards scMR1-Fc-H17R (corresponding to the MR1*02 allele). B. Binding of a self-reactive MAIT (E8) TCR to the same sensor chip containing the immobilised scMR1-Fc constructs, indicating that all scMR1 constructs are functional and loaded to a similar level. C. The MC.7.G5 TCR does not bind to refolded MR1-5-OP-RU, MR1-6-FP or MR1-5-FSA, representing a range of the known MR1-ligand complexes. All the MR1-ligand complexes are functional as shown by binding of either the VT001 MAIT TCR or the self-reactive MAIT (E8) TCR to the same sensor chip containing the immobilised refolded MR1-ligand complexes. Dissociation constants (K_D_) calculated at equilibrium, mean ± SD. All titrations were repeated at least in duplicate in independent experiments, one representative titration shown.

**Supplementary Figure 4**


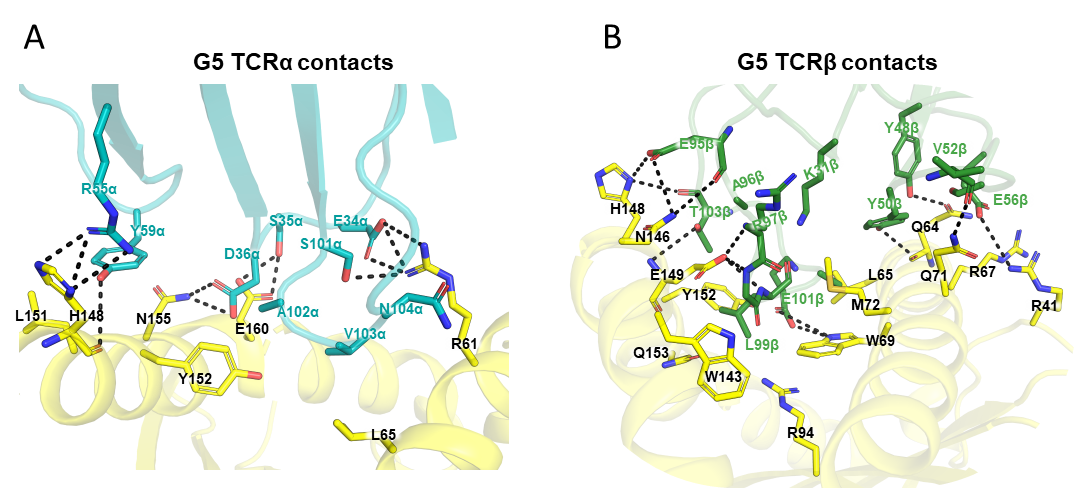


**Figure S4. Interactions between G5 TCR and MR1 R9H complex.** A. MC.7.G5 TCR alpha interactions with MR1 R9H. Residues within 4Å at the interface are shown as sticks. The dotted lines indicate polar contacts. B. G5 TCR between interactions with MR1 R9H.
